# Characterization of DoTc2 4510—Identifying HPV16 Presence in a Cervical Carcinoma Cell Line Previously Considered to Be HPV-Negative

**DOI:** 10.3390/cancers15153810

**Published:** 2023-07-27

**Authors:** Nika Vučković, Karin Hoppe-Seyler, Angelika B. Riemer

**Affiliations:** 1 Immunotherapy and Immunoprevention, German Cancer Research Center (DKFZ), 69120 Heidelberg, Germany; 2 Molecular Vaccine Design, German Center for Infection Research (DZIF), Partner Site Heidelberg, 69120 Heidelberg, Germany; 3 Faculty of Biosciences, Heidelberg University, 69120 Heidelberg, Germany; 4 Molecular Therapy of Virus-Associated Cancers, German Cancer Research Center (DKFZ), 69120 Heidelberg, Germany

**Keywords:** DoTc2 4510, cervical cancer, HPV, HPV-negative cervical cancer, cell authentication

## Abstract

**Simple Summary:**

Cervical cancer is one of the most deadly cancers for women worldwide. Its incidence is, in virtually all cases, causally tied to a human papillomavirus (HPV) infection that had not been cleared by the immune response of the host. There is a small subset of cervical cancers, however, that do not show evidence of HPV presence, thus representing a rare gynecological cancer entity. While HPV-related cervical cancer is studied extensively, HPV-independent cervical cancer studies are lacking, together with appropriate models. We obtained a cervical cancer cell line, DoTc2 4510, described in the literature as HPV-negative. After suspicous preliminary results showing HPV presence, we proceeded to characterize the cell line in detail, proving it is truly HPV-transformed. With this publication, we aim to inform the community about the HPV status of the studied cell line, as well as emphasize the importance of stringent controls in cancer model establishment, especially for rare cancers.

**Abstract:**

Cervical cancer is the fourth leading cause of cancer deaths in women, with over 340,000 women dying from this disease in 2020. Almost all cases have an underlying persistent infection with an oncogenic high-risk type of human papillomavirus (HPV), mainly HPV16. While cervical squamous cell carcinoma is hardly ever HPV-negative, a small subset of adenocarcinoma exhibits absence of HPV, even after disproval of false-negative testing results due to low viral load. This proportion is evident in many cervical cancer studies and is reflected in the repertoire of model cell lines commonly used in research. As the viral origin of cervical cancer makes it a disease preventable and potentially treatable by immunotherapeutic approaches, it is the focus of many studies. For pertinent research, both a broad set of HPV-infected cervical carcinoma models are required, as well as stringent negative controls. A ubiquitously used HPV-negative cervical adenocarcinoma cell line is C-33A. Another cervical cancer cell line is available for purchase from the American Type Culture Collection (ATCC), namely DoTc2 4510, described to be HPV-negative and thus as a model for a rare gynecological malignancy. Here, we present findings proving that DoTc2 4510 is, in fact, an HPV16-positive cell line. This we assessed using a highly sensitive nested multiplex PCR protocol adapted for the identification of 12 carcinogenic HPV types and a second PCR targeting the HPV16 oncogenes E6 and E7. Subsequently, the protein expression of E6 and E7 was examined, as well as the expression of their target proteins p53, p21, and p16^INK4a^, to assess E6/E7 functionality. Finally, to attest to the survival dependence of DoTc2 4510 cells on HPV16, we performed an HPV16 E6/E7-targeted siRNA knock-down, which indeed led to senescence induction. Together, these findings demonstrate that DoTc2 4510 is an HPV16-transformed cell line.

## 1. Introduction

Cervical cancer (CxCa) is considered to be the fourth leading cause of cancer-related death in women worldwide, with around 340,000 women dying of CxCa only in 2020 [1]. Nearly all CxCa cases are attributed to persistent human papillomavirus (HPV) infection with one of the oncogenic high-risk HPV types, such as HPV16, while there is only a small fraction of HPV-independent disease [2]. When divided by histological characteristics, CxCa can be squamous cell carcinoma (SCC), adenocarcinoma (AC), or adenosquamous carcinoma (ASC). SCC is the globally most common histological variant, accounting for around 70% of CxCa. This is reflected in studies where SCC is predominantly researched, and AC and ASC are usually grouped together [3]. Infection with high-risk HPV types is strongly causally linked to SCC, with HPV DNA being identified in almost 100% of all SCC cases. No definitively HPV-free SCC has been reported [4]. Meanwhile, AC is associated with HPV in 62–100% of cases, and research has shown that nearly 68% of cervical cancers appointed to be HPV-free had been falsely diagnosed as negative for HPV [4,5]. Thus, HPV-negative cervical cancers can be considered to be rare gynecological cancers.

While the carcinogenesis of HPV-positive cervical cancer is well understood, much less is known about HPV-negative cancer development. The mechanism of carcinogenesis deployed by high-risk HPV is through the E6 and E7 oncoproteins. They do so by dysregulating the cell cycle by inhibiting/degrading the tumor suppressor proteins p53 and Retinoblastoma protein (pRb). The E7 oncoprotein interferes with the normal transcription complex containing pRb. E7 binds to pRb, blocking it, thus disturbing the cell cycle and driving the cell from the G1 into the S phase. With pRb inactive, downstream tumor suppressor protein p16^INK4a^ expression is upregulated. Simultaneously, the E6 oncoprotein binds to p53 and consequently leads to its destruction [6]. The combined effects of E6 and E7 on p53 and pRb also influence the expression of p21, a subsequent member of both signaling pathways, and inhibit its tumor suppressor function [7]. On the other hand, HPV-negative cancer is thought to be driven by mutations in tumor-associated genes, including TP53, PIK3CA, and CDKN2A [4].

Research on the rare HPV-independent cervical cancer is limited in both scope and appropriate models. To understand the mechanism of HPV-independent cervical carcinogenesis, there is a pressing demand for appropriate models to study [8]. A ubiquitously used HPV-negative cervical cancer cell line is C-33A, an epithelial cell line that was derived from an invasive cervical carcinoma [9,10]. This cell line is utilized as a negative control cell line in HPV-positive cervical cancer studies and for studies on HPV-negative cervical cancer [11]. The relative incidence of HPV-induced and HPV-independent CxCa is reflected in the available cell lines, and aside from C-33A, there are no easily available HPV-negative cervical carcinoma cell lines. However, the American Type Culture Collection (ATCC) offers another cell line described to be HPV-negative, namely DoTc2 4510.

At the time of starting a new project, thorough characterization and authentication of model cell lines is recommended [12]. Following this principle, the DoTc2 4510 cell line was assessed upon purchase and introduction into our laboratory and kept in quarantine until results were received. We analyzed the cells in detail by authentication, examined them for possible contaminations, identified the HLA-type, and then proceeded to check for any trace of HPV on the DNA, RNA, and protein levels. As the presence of HPV16 was identified on all tested levels, we further investigated the effect of the detected HPV oncogenes by analyzing downstream cell cycle control proteins as well as cell viability upon knock-down of the HPV oncogenes.

## 2. Materials and Methods

### 2.1. Cell Culture

This work included four cell lines, HPV-negative normal oral keratinocytes, NOK (kindly provided by Karl Munger, Tufts University School of Medicine, Boston, MA, USA), HPV16-positive cervical cancer cell line CaSki (CRM-CRL-1550, ATCC, Manassas, VA, USA), HPV-negative cervical cancer cell line C-33 A (HTB-31, ATCC) and the cervical cancer cell line DoTc2 4510 (CRL-7920, ATCC). NOK was cultured in Keratinocyte SFM (1×) supplemented with Human Recombinant EGF, Bovine Pituitary Extract (Gibco, Paisley, UK), and 10% Fetal Bovine Serum (FBS). CaSki, C-33 A, and DoTc 2 4510 were grown in DMEM (Gibco) supplemented with 10% FBS, 1% Penicillin/Streptomycin, and 1% L-Glutamine.

### 2.2. Authentication, Contamination Testing, HLA Typing

Upon delivery of DoTc2 4510, total DNA extraction was performed using the QIA-amp DNA Mini Kit (QIAGEN, Hilden, Germany) according to the manufacturer’s instructions, and DNA was prepared following testing service providers ‘requests. Cells were put in culture in quarantine until results from standard routine cell authentication and contamination tests were received. Both tests were performed by Multiplexion GmbH (Heidelberg, Germany). The cell line was authenticated by determining genetic characteristics via STR-profiling technology, while contamination testing was run against 14 Mycoplasma species, Epstein–Barr virus, Squirrel Monkey Retrovirus, and 12 contaminating cell lines. HLA-typing was performed by Deutsche Knochenmarkspenderdatei (DKMS) Life Science Lab GmbH (Dresden, Germany). HLA typing results are provided in accordance with the current HLA nomenclature as G codes, NMDP codes (MAC), or 2-field allele names. NMDP (MAC) codes can be decoded under https://hml.nmdp.org/MacUI/ (accessed on 20 June 2023). CaSki, NOK, and C-33 A were previously examined in the same manner.

### 2.3. Nested Multiplex PCR for Type-Specific HPV Detection

Total DNA extraction was as described above. Nested multiplex PCR (NM-PCR) was performed following a published protocol [13]. In brief, 125 nanograms of total isolated DNA was used in the first round of amplification, carried out with a single forward consensus general primer (GP) GP-E6-3F 5′ GGG WGK KAC TGA AAT CGG T 3′ and two consensus reverse primers, GP-E7-5B 5′ CTG AGC TGT CAR NTA ATT GCT CA 3′ and GP-E7-6B 5′ TCC TCT GAG TYG YCT AAT TGC TC 3′. These primers target a sequence encompassing both oncogenes for HPV E6 and E7 of several HPV types. Initial denaturation was at 94 °C for four minutes, followed by 40 cycles of denaturation at 94 °C for one minute, annealing at 40 °C for one minute, elongation at 72 °C for two minutes, and final elongation at 72 °C for 10 min. Two microliters of the resulting PCR product were used in the second round carried out by HPV-type-specific nested multiplex primers for HPV types 16, 18, 31, 33, 35, 39, 45, 51, 52, 56, 58, 59 (Appendix A Table A1). Initial denaturation was at 94 °C for four minutes, succeeded by 35 cycles of denaturation at 94 °C for 30 s, annealing at 56 °C for 30 s, elongation at 72 °C for 45 s, and final elongation at 72 °C for four minutes. In both rounds, all reactions were carried out in a final volume of 50 microliters consisting of 25 microliters of REDTaq^®^ ReadyMix PCR-Reaction mix and 15 picomols of each primer (all Sigma-Aldrich, Taufkirchen, Germany). 

### 2.4. PCR Detection of HPV16 E6/E7

The same extracted total DNA was subsequently also inspected for the presence of HPV16-specific E6 and E7 gene sequences adhering to a protocol shared by Tarik Gheit (Word Health Organization). Shortly, amplification was performed using forward 5′CGA AAC CGG TTA GTA TAA 3′ and reverse 5′ GTA TCT CCA TGC ATG ATT 3′ primers to detect E6, and forward 5′ ATA ATA TAA GGG GTC GGT GG 3′ and reverse 5′ CAT TTT CGT TCT CGT CAT CTG 3′ for E7. PCR conditions were initial denaturation at 95 °C for 15 min, then 35 cycles of denaturation at 94 °C for one minute, annealing at 55 °C for one minute, elongation at 72 °C for two minutes, and final elongation at 72 °C for 10 min. Both reactions were performed in a total volume of 50 microliters containing 25 microliters of of REDTaq^®^ ReadyMix PCR-Reaction mix and 25 picomol of each primer (all Sigma-Aldrich).

### 2.5. Gel Electrophoresis and Product Sequencing

Ten microliters of each amplification product were analyzed by electrophoresis on a 2% agarose gel stained with GelRed^®^ Nucleic Acid Stain 10,000× (Biotrend, Cologne, Germany) and photographed with INTAS Gel capture. Furthermore, products were cleaned up using NucleoSpin Gel and PCR Clean-up (Macherey-Nagel, Düren, Germany) according to the manufacturer’s instructions and sequenced (Microsynth AG, Balgach, Switzerland). Sequence alignment was performed using Human papillomavirus type 16, complete genome, GenBank: K02718.1.

### 2.6. Western Blot

For protein expression analysis by Western blot, cells were lysed using lysis buffer consisting of 10 mM tris-HCL pH 7.5, 50 mM KCl, 2 mM MgCl_2_, 1% Triton X-100, 1 mM DTT, 1 mM PMSF, and protease inhibitor cocktail (Roche, Basel, Switzerland). Samples were incubated for 20 min on ice with periodic mixing followed by 10 min centrifugation at 14,000× *g* at 4 °C. Proteins were mixed with 4× Laemmli-buffer-containing β-mercaptoethanol and then boiled at 95 °C for 5 min. Isolated proteins were separated by SDS-PAGE and then transferred to a PVDF membrane using semi-dry transfer. Membranes were blocked followed by overnight incubation at 4°C with primary antibodies (HPV16 E6 E6-6F4 (Euromedex, Souffelweyersheim, France), HPV16 E7 NM2 (in-house produced), p21 Waf1/Cip1 (F-5), P16INK4A (DCS-50)sc-65476, p53 (DO-1)sc-126 (Santa Cruz Biotechnology, Dallas, Texas), β-actin AC-74 (Sigma-Aldrich), GAPDH (GT239) (Biozol, Eching, Germany). HRP-coupled secondary antibodies (IgG anti-Mouse IgG (H+L)-HRPO (Dianova, Hamburg, Germany) were then incubated with the membrane for 1 h. After adding the chromogenic substrate ECL™ Prime Western Blotting detection reagents (Cytiva Europe GmbH, Freiburg im Breisgau, Germany), a chemiluminescence signal was observed in a Biorad Chemiluminescence Detector (BioRad ChemiDoc, Hercules, CA, USA).

### 2.7. siRNA Transfection

Synthetic siRNAs (Silencer Select, Life Technologies, Carlsbad, CA, USA) were transfected with Lipofectamine RNAiMAX (Invitrogen, Waltham, MA, USA), according to the manufacturer’s instructions to a final siRNA concentration of 10 nM. Three different siRNAs blocking HPV16 E6/E7 oncogene expression were pooled at equimolar concentrations to minimize potential off-target effects, as detailed previously [14]. The siRNA target sequences were the following: 16E6/E7-1: 5′-CCGGACAGAGCCCAUUACA-3′; 16E6/E7-2: 5′-CACCUACAUUGCAUGAAUA-3′; 16E6/E7-3: 5′-CAACU-GAUCUCUACUGUUA-3′; siNeg: 5′-TACGACCGGTCTATCGTAG-3′.

### 2.8. Colony Formation Assay

For colony formation assays, DoTc2 4510 cells were transfected with siRNA, as described above. Three days after the transfection, cells were reseeded and incubated for 11 days. Later, cells were fixed with formaldehyde and stained with crystal violet, as previously described [15,16].

### 2.9. Senescence Induction Assay

DoTc2 4510 were seeded three days after siRNA transfection and grown for 4 days.

Then, cell morphology was examined for typical characteristics of senescence (enlargement, flattening, protrusions), and senescence-associated β-galactosidase activity was assessed, as previously described [15,16].

## 3. Results

### 3.1. Authentication, Contamination Testing, HLA Typing

Authentication testing by STR-Profiling confirmed the cell line is indeed DoTc2 4510, and contamination testing showed no presence of any of the examined-for entities. HLA-typing results showed DoTc2 4510 cells to be homozygous or hemizygous (potentially after loss of heterozygosity) for both HLA class-I and HLA-class-II, carrying A*03:01:01, B*55:01:01, C*03:DJUJV, DRB1*03:RPXT, DQB1*02:CGRKD, DQB1*02:CGRKD, DPB1*10:AWFCR. (Letters represent NMDP (MAC) codes; see Methods for decoding information.) The HLA class-I allotype is the same as previously reported for this cell line [17]. 

### 3.2. HPV Detection and Genotype Identification by Nested Multiplex PCR and HPV16 E6/E7 Specific PCR

**Figure 1:**
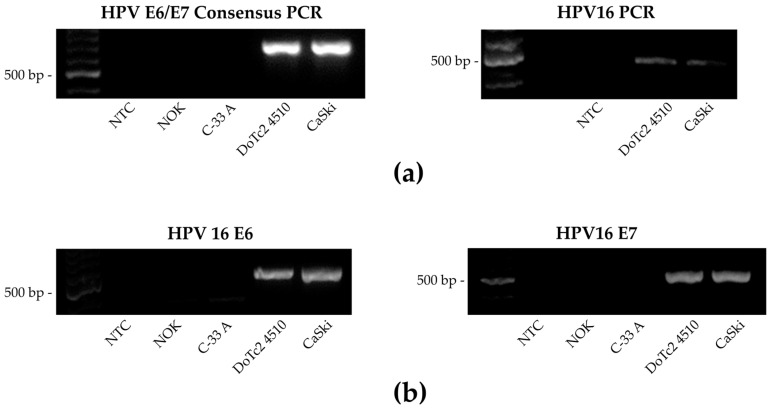
HPV detection and genotype identification by two separate PCR protocols: Nested multiplex PCR (NM-PCR) and HPV16 E6/E7 specific PCR. NOK: normal oral keratinocytes, negative control; C33A: HPV-negative cervical adenocarcinoma cell line; DoTc2 4510: cervical cancer cell line, investigated; CaSki: HPV16-positive cervical cancer cell line, positive control (**a**) Left: First round of NM-PCR. PCR products were generated with general primers GP-E6-3F and GP-E7-5B/6B (630 bp). Right: Second round of NM-PCR. The presence of distinct 12 carcinogenic HPV types was tested for each positive cell line using the first round PCR product. (**b**) HPV16 E6/E7 specific amplification. Detection of HPV16 was attested via separate HPV16 E6 (524 bp) and HPV16 E7 (505 bp) PCR assays. Uncropped gel images are presented in Figure S1.

After confirming the identity of DoTc2 4510, cells were cultured and examined for HPV to confirm they were HPV-negative. Nested multiplex PCR, testing for the potential presence of 12 high-risk HPV types, showed the presence of HPV16 (Figure 1a). PCR analysis with primers specific for HPV16 E6 and E7 corroborated the original finding of HPV16 DNA presence (Figure 1b). PCR products of DoTc2 4510 from both amplification protocols were sequenced and aligned perfectly with the reference genome for human papillomavirus type 16. These results show that DoTc2 4510 is positive for HPV16 DNA.

### 3.3. Expression of HPV16 E6 and E7 Oncoproteins and Affected Downstream Tumor Suppressor Proteins

**Figure 2:**
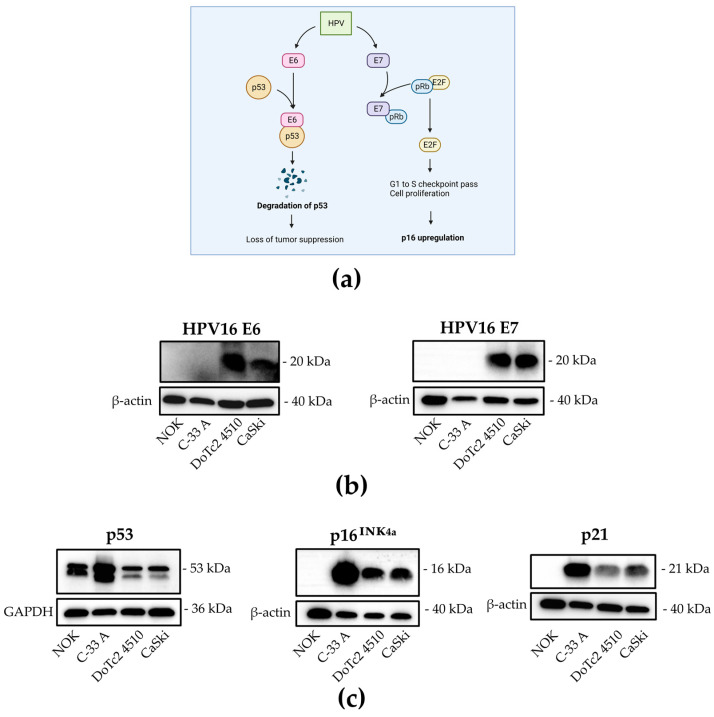
Expression of HPV16 E6 and E7 oncoproteins and affected downstream tumor suppressor proteins. (**a**) Schematic depiction of viral oncoprotein effects on cell cycle control proteins adapted from Wai 2020. (**b**,**c**) Cellular lysate of each indicated cell line was analyzed for expression of E6 and E7 (**b**) and p53, p16, and p21 (**c**). GAPDH and β-actin were used as loading controls. Original Western blots are presented in Figure S2.

To determine if HPV16 E6 and E7 are present on a translational level, protein expression was examined by Western blot (Figure 2). Figure 2a shows a schematic representation of the main effects of E6 and E7 on cell cycle control proteins in HPV-transformed cells. Both HPV16 oncoproteins E6 and E7 were detected (Figure 2b), as well as the expected effects on the analyzed downstream proteins, namely p53 for E6 and p16 for E7 and p21 as an element of both p53 and pRb signaling cascades. All of the proteins tested for show expression levels as observed and expected in HPV-positive cancer cells, which are downregulation of p53, upregulation of p16, and downregulation of p21 (Figure 2c). These results show that HPV16 E6 and E7 oncoproteins are translated in DoTc2 4510, they are functional and affect tumor suppressor proteins as characteristic for HPV16 positive cells.

### 3.4. Assessment of DoTc 2 4510 Cells’ Dependence on HPV16 E6 and E7 Oncoproteins by siRNA Knock-Down

**Figure 3:**
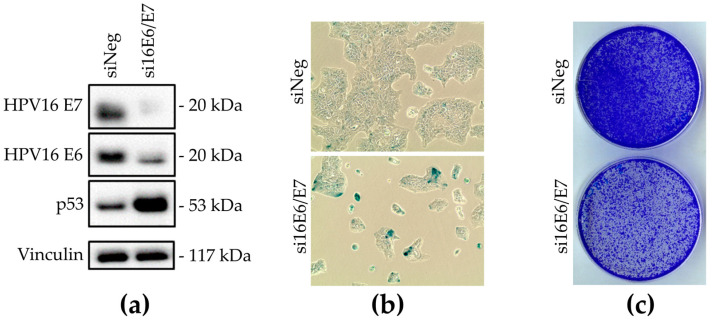
Assessment of DoTc2 4510 cell dependence on HPV16 E6 and E7 oncoproteins by siRNA knock-down. (**a**) Expression of HPV16 E6, E7, and p53 analyzed by Western blot after siRNA knock-down. siNeg: siRNA knock-down negative control, Vinculin, loading control. (**b**) Analysis of cell morphology and detection of senescence-associated β-galactosidase activity after transfection with non-targeting siRNA (siNeg) and HPV16 E6/E7-targeting (siE6/E7) siRNA pools. (**c**) Colony formation capacity of DoTc2 4510 cells was analyzed after transfection with non-targeting siRNA and HPV16 E6/E7-targeting siRNA pools. Original Western blots are presented in Figure S3.

With HPV16 DNA presence and HPV16 oncoprotein expression confirmed, the next step was to examine if DoTc2 4510 cells are dependent for survival on the HPV16 oncoproteins, which is another typical feature of HPV16-transformed cells (Figure 3). HPV16 E6/E7-targeting siRNAs indeed decreased expression of the HPV16 E6 and E7 oncoproteins while consequently increasing the quantity of detectable p53 (Figure 3a). Upon morphological analysis of HPV16 E6/E7-targeting siRNA-transfected cells, the majority displayed the typical features of senescent cells, like flattening and enlargement. Staining for senescence-associated β-galactosidase further affirmed the presence of senescent cells (Figure 3b). Moreover, cells showed a marked decline in colony formation (Figure 3c). These findings indicate that DoTc2 4510 cells indeed enter a permanent cell cycle arrest when HPV oncogenes are knocked down.

## 4. Discussion

HPV-negative cervical cancer represents a rare disease entity. An indispensable prerequisite for constructive research comparing HPV-positive and HPV-negative cervical cancers is good and appropriate in vitro models. Cancer cell lines representing both subtypes of the disease, as well as stringent negative controls for virus-related research, are crucial. Alertness is particularly important in working with models of rare disease subtypes due to the increased difficulty of obtaining material that was accurately classified at the time of patient diagnosis. In the process of diagnostics, HPV-negative results are often false negatives due to differences in sensitivity in approved and used detection procedures [4]. More rigorous testing methods need to be used in order to correctly assess HPV status, especially in cases of CxCa seemingly HPV-independent. Such approaches include performing highly sensitive PCR, stringent histological analyses, and diligent sampling. This would allow more pertinent treatment as well as the generation of truthful cell line models. 

DoTc2 4510 is available for purchase from ATCC as a component of the cervical cell line panel. Disclosed is scarce information on the characterization of the cells, limited to the description of the cell line as epithelial and originating from a female with cervical carcinoma. In some of the referenced papers, HPV status is not reported, while several studies used and published DoTc2 4510 as an HPV-negative cell line, some of them grouping it to SCC while others refer to it as AC [18,19,20,21,22,23,24]. In fact, there are only two publications mentioning DoTc2 4510 as HPV-positive, which corroborates our findings. The first study detected HPV16 E6 on the DNA level [25], while the second detected transcripts of all HPV16 viral genes by RNA sequencing [17]. However, in these studies, the cell line was not analyzed in depth, especially not for viral protein expression and functionality.

The focus of our group is developing a therapeutic HPV vaccine by detecting HPV target epitopes using sensitive mass spectrometry methods requiring stringent controls. We thus acquired the DoTc2 4510 cell line to use it as an additional HPV-negative control cervical cancer cell line. For this reason, as a first step, we applied our routine highly sensitive nested multiplex PCR (NMPCR), which was developed to detect even low levels of 12 high-risk HPV types [13] to confirm assumed HPV absence. Upon positive results in both the NMPCR and an additional HPV16 oncogene-specific PCR on the genome level and inconclusive HPV status description in subsequent literature research, we continued to investigate the cell line in depth to characterize it. Expression of HPV16 oncoproteins and their typical effects on targeted cellular proteins was clearly shown. Furthermore, we demonstrated that DoTc2 4510 are HPV-oncogene-addicted and thus show all the hallmarks of being HPV16-transformed. 

The fact that DoTc2 4510 is widely accepted as HPV-negative in the scientific community may stem from two circumstances: ATCC describes cell lines on their website as submitted by the depositor, and customers tend not to question descriptions found on the ATCC website. We want to stress again that the cell line is not indicated to be either HPV-negative or HPV-positive on the ATCC website but just described by references [26].

The case of DoTc2 4510 highlights the importance of good scientific practices, especially in regard to the diligent management of experimental material. This is particularly important when working with cell lines that supposedly stem from rare oncologic entities, such as HPV-negative cervical cancer. There is a problem recognized within the scientific community of erroneously published data and conclusions due to research performed on inappropriate and irrelevant material. This is visible in the lack of reproducibility and paper retractions, which have been the subject of several publications [27,28]. The significance of regular cell line authentication and contamination tests has also been emphasized in many papers and is imperative for high-quality research [29,30].

## 5. Conclusions

In this study, we have characterized the DoTc2 4510 cervical carcinoma cell line. Our data show that DoTc2 4510 cells are, in fact, HPV16-transformed, with evidence on genomic, translational, and functional levels. These findings exclude them from the HPV-negative cervical carcinoma subtype, a rare gynecological cancer that is in great need of representative models and comprehensive studies. Another inference of this work lies in highlighting the importance of routine cell authentication and a conscientious approach in conducting cell line-based studies, as inadequately designated cell lines seriously harm research on a fundamental level.

## Data Availability

All data is contained within the article.

## Supplementary Materials

The following supporting information can be downloaded at: https://www.mdpi.com/article/10.3390/cancers15153810/s1, Figure S1. Whole gel images of HPV detection and genotype identification by two separate PCR protocols: Nested multiplex PCR (NMPCR) using general primers GP-E6/E7 (a) and HPV 16 E6/E7 specific PCR (b). Figure S2. Whole Western blot images of expression analysis of HPV 16 E6 and E7 oncoproteins and affected downstream tumor suppressor proteins. Figure S3. Whole Western blot images of expression analysis of HPV16 E6 and E7 oncoproteins and p53 after siRNA knock-down.

## Appendix A

**Table A1:** HPV primers used in second step of NM-PCR as described by Sotlar et al. [13]. Annoted are HPV types, amplicon size, forward (top), and reverse primer (bottom).

HPV Type	Amplicon (bp)	Sequence (5′-3′)
16	457	CAC AGT TAT GCA CAG AGC TGC
		CAT ATA TTC ATG CAA TGT AGG TGT A
18	322	CAC TTC ACT GCA AGA CAT AGA
		GTT GTG AAA TCG TCG TTT TTC A
31	263	GAA ATT GCA TGA ACT AAG CTC G
		CAC ATA TAC CTT TGT TTG TCA A
33	398	ACT ATA CAC AAC ATT GAA CTA
		GTT TTT ACA CGT CAC AGT GCA
35	358	CAA CGA GGT AGA AGA AAG CAT C
		CCG ACC TGT CCA CCG TCC ACC G
39	280	GAC GAC CAC TAC AGC AAA CC
		TTA TGA AAT CTT CGT TTG CT
45	151	GTG GAA AAG TGC ATT ACA GG
		ACC TCT GTG CGT TCC AAT GT
51	223	GAG TAT AGA CGT TAT AGC AGG
		TTT CGT TAC GTT GTC GTG TAC G
52	229	TAA GGC TGC AGT GTG TGC AG
		CTA ATA GTT ATT TCA CTT AAT GGT
56	181	GTG TGC AGA GTA TGT TTA TTG
		TTT CTG TCA CAA TGC AAT TGC
58	274	GTA AAG TGT GCT TAC GAT TGC
		GTT GTT ACA GGT TAC ACT TGT
59	215	CAA AGG GGA ACT GCA AGA AAG
		TAT AAC AGC GTA TCA GCA GC
