# Supplementary material for: Characterization of DoTc2 4510—Identifying HPV16 Presence in a Cervical Carcinoma Cell Line Previously Considered to Be HPV-Negative
Source: Cancers (Basel). 2023 Jul 27;15(15):3810. doi: 10.3390/cancers15153810 (PMC10417116; doi:10.3390/cancers15153810)

Supporting information

Figure S1. Whole gel images of HPV detection and genotype identification by two separate PCR protocols: Nested multiplex PCR (NMPCR) using general primers GP-E6/E7 (a) and HPV16 E6/E7 specific PCR (b).

(a)

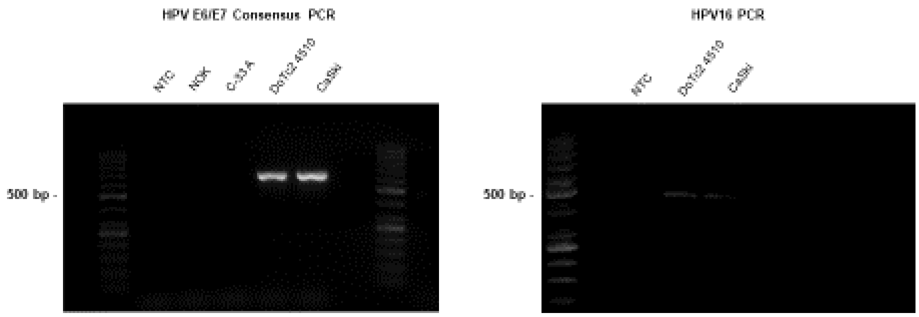

(b)

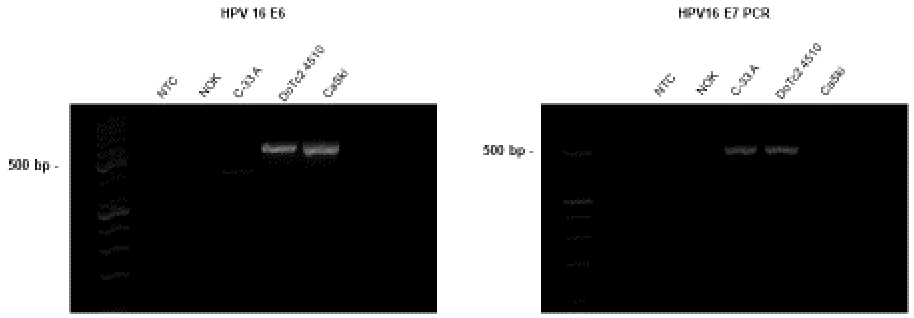

Figure S2. Whole Western blot images of expression analysis of HPV16 E6 and E7 oncoproteins and affected downstream tumor suppressor proteins.

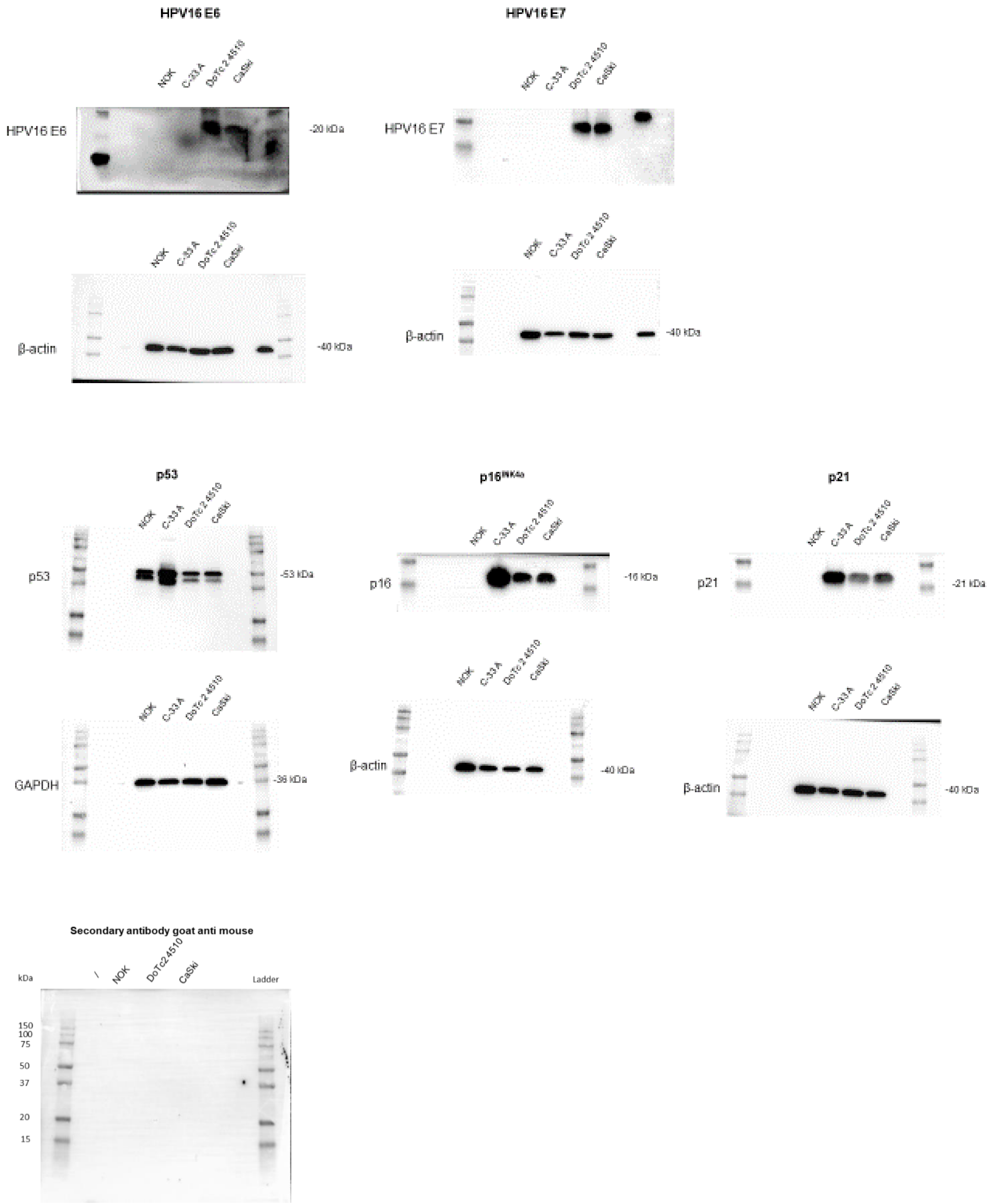

Figure S3. Whole Western blot images of expression analysis of HPV16 E6 and E7 oncoproteins and p53 after siRNA knock-down. This figure relates to main Figure 3a. Please note that the blots were imaged with another device than in Figures 2/S2, which is why they appear inverted here.

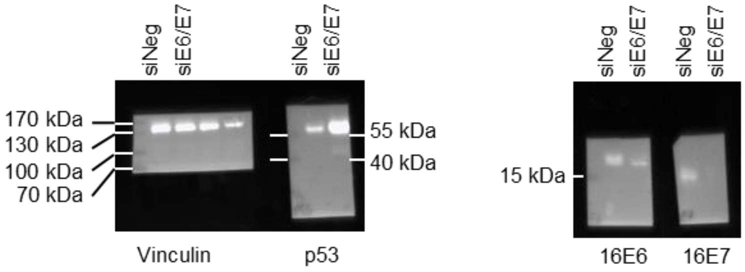

Supplement: Supplementary file 1 [file cancers-15-03810-s001.zip › cancers-2508620-supplementary.pdf]
